# Multiple Medication Adherence and Related Outcomes in Community-Dwelling Older People on Chronic Polypharmacy: A Retrospective Cohort Study on Administrative Claims Data

**DOI:** 10.3390/ijerph19095692

**Published:** 2022-05-07

**Authors:** Carlotta Franchi, Monica Ludergnani, Luca Merlino, Alessandro Nobili, Ida Fortino, Olivia Leoni, Ilaria Ardoino

**Affiliations:** 1 Laboratory of Pharmacoepidemiology and Human Nutrition, Department of Health Policy, Istituto di Ricerche Farmacologiche Mario Negri IRCCS, Via Mario Negri 2, 20156 Milan, Italy; alessandro.nobili@marionegri.it (A.N.); ilaria.ardoino@marionegri.it (I.A.); 2 Direzione Sanitaria—Centro Cardiologico Monzino (I.R.C.C.S.), 20138 Milan, Italy; monicluder@gmail.com (M.L.); luca.merlino@cardiologicomonzino.it (L.M.); 3 Directorate General for Health, Lombardy Region, 20124 Milan, Italy; ida_fortino@regione.lombardia.it (I.F.); olivia_leoni@regione.lombardia.it (O.L.)

**Keywords:** drug adherence, drug use, polypharmacy, administrative database, real-world evidence, frail elderly, patient outcome assessment

## Abstract

Poor medication adherence compromises treatment efficacy and adversely affects patients’ clinical outcomes. This study aims to assess (1) multiple medication adherence to the most common drug classes chronically prescribed to older people, (2) the factors associated, and (3) the clinical outcomes. This retrospective cohort study included 122,655 community-dwelling patients aged 65–94 years old, newly exposed to chronic polypharmacy, and recorded in the Lombardy Region (northern Italy) administrative database from 2016 to 2018. Multiple medication adherence was assessed for drugs for diabetes, antithrombotics, antihypertensives, statins, and bisphosphonates, by calculating the daily polypharmacy possession ratio (DPPR). One-year mortality, nursing home, emergency department (ED), and hospital admission rates were calculated for 2019. The most prescribed drugs were antihypertensives (89.0%). The mean (std.dev) DPPR was 82.9% (15.6). Being female (OR = 0.85, 95%CI: 0.84–0.86), age ≥85 years (OR = 0.77, 95%CI: 0.76–0.79), and multimorbidity (≥4 diseases, OR = 0.88, 95%CI: 0.86–0.90) were associated with lower medication adherence. A higher DPPR was associated with clinical outcomes—in particular, improved survival (HR = 0.93 for 10/100-point increase, 95%CI: 0.92–0.94) and lower incidence in nursing home admissions (SDHR = 0.95, 95%CI: 0.93–0.97). Adherence to the most common chronic drugs co-prescribed to the older population was high. Better multiple medication adherence was associated with better clinical outcomes.

## 1. Introduction

Patients’ adherence to their medications is crucial in the management of their pharmacological treatment for reaching clinical goals. Adherence is defined by the World Health Organization as “the extent to which the person’s behavior (including medication-taking) corresponds with agreed recommendations from a healthcare provider” [1].

People aged 65 years or more commonly suffer chronic and multiple concomitant diseases, that often expose them to a multiple drug regimen (polypharmacy) [2,3,4]. 

Medication adherence for older adults prescribed polypharmacy can be particularly challenging, and previous studies have indeed shown that increases in both the number of prescribed medications and the regimen complexity correlate with lower medication adherence [1,5,6,7,8,9]. In developed countries, adherence among patients suffering from chronic diseases averages only 50% [1] and is, therefore, a widespread problem of striking magnitude. Poor medication adherence severely compromises the effectiveness of treatments, contributing to increased morbidity, mortality, and healthcare costs [10]. Thus, the assessment of adherence to polypharmacy in older populations is clinically relevant and crucial for policymakers, researchers, and healthcare professionals alike, but it imposes a growing need for robust estimates. The literature introduces several measures depending on data and economic resource availability. An administrative database can assist stakeholders in this evaluation. When using administrative claims data, several measures are proposed to calculate adherence to a single drug. Those most frequently used are the medication possession ratio (MPR), the proportion of days covered (PDC), or medication refill adherence (MRA), which provide almost the same results and are usually recommended for their simplicity and the small number of data required [11]. However, the high prevalence of polypharmacy, especially in older populations, demands a composite measure to assess multiple medication adherence as a whole and to assess the overall effect of medication adherence on a patient’s clinical outcomes, providing reliable and unbiased results. 

Until now, no standard measure has been proposed to assess multiple medication adherence, and it is usually calculated as an average of the indices previously mentioned for single drugs, though this often provides biased estimates. Few attempts have been made to calculate adherence to multiple concurrent medications with robust and validated methods, such as the daily polypharmacy possession ratio (DPPR). The DPPR estimates the average number of medications available for use on each day of the observation period [12,13].

With this background, and considering the gaps in our knowledge, the aims of this study were to assess (1) multiple medication adherence to five of the most common chronic drug classes prescribed to community-dwelling older people, using the DPPR, (2) the factors associated with multiple medication adherence, and (3) the patients’ clinical outcomes (mortality, nursing home, number of emergency department and hospital admissions). 

## 2. Materials and Methods 

### 2.1. Study Design

Data for the present study were obtained from the Healthcare Administrative Database of the Lombardy Region, which stores information for all residents living in the region (nearly ten million inhabitants, around 16% of the Italian population). The administrative database collects outpatient (demographic data, drugs dispensed, specialist visits, diagnostic procedures, and laboratory tests) and inpatient healthcare data (hospitalizations reporting therapeutic procedures and the main diagnoses at discharge) provided by public and private accredited providers and totally or partially funded by the National Health Service (NHS). More details on the structure of this database have been provided elsewhere [5]. The database is routinely updated for administrative and reimbursement purposes. Each beneficiary of the NHS is assigned a unique identification code, which allows record linkage among different data sources. According to current Italian law on patient privacy, the unique identification code is automatically converted to an anonymous code.

All drugs were classified according to the Anatomical Therapeutic Chemical (ATC) Classification recommended by the World Health Organization. 

### 2.2. Participants and Setting

In this retrospective cohort study, we selected community-dwelling patients aged 65–94 years old in the Lombardy Region and newly exposed to chronic polypharmacy from 1 January to 31 December 2016. Chronic polypharmacy was defined as “the dispensing of five or more drugs during one month for at least six months (consecutive or not) in a year” [4,5,14]. Patients exposed to chronic polypharmacy in the previous 12 months were excluded. All drug subgroups identified at the IV ATC level prescribed by general practitioners (GPs) and dispensed through local pharmacies and entirely or partially reimbursed by the NHS were considered for identifying polypharmacy, regardless of whether they were prescribed for the long term, for acute conditions, or on demand. The overall period of polypharmacy assessment ended on 31 December 2017. 

### 2.3. Assessment of Medication Adherence 

Adherence was assessed for the following five drug classes, which are among those most commonly prescribed to older patients for long-term clinical conditions:

(1) Drugs for diabetes, excluding insulins and analogs (ATC: A10B-A10X);
(2) Antithrombotic agents, excluding Vitamin K antagonists and heparin (ATC: B01AC-B01AX);
(3) Agents acting on the renin–angiotensin system (angiotensin-converting enzyme (ACE)-inhibitors and angiotensin II receptor antagonists), hereafter referred to as antihypertensives (ATC: C09);
(4) Lipid-modifying agents, referred to as statins (ATC: C10);
(5) Drugs for the treatment of bone diseases, mainly bisphosphonates (ATC: M05B).

Adherence was calculated for each drug class and for the multiple medication regimens to which a patient was exposed. The observation window ranged from the date on which the first drug class was dispensed (index date), during the period in which the patient was exposed to chronic polypharmacy, up to 31 December 2018. Thus, the overall period of adherence assessment could range from 1 January 2016 to 31 December 2018. Patients who died, were admitted to nursing homes, migrated, or were hospitalized more than 12 times during the period of adherence assessment (i.e., from 1 January 2016 to 31 December 2018) were excluded. Patients first prescribed the drugs of interest after 1 January 2018 were also excluded, in order to ensure the assessment of medication adherence for at least one year. For each drug category, at least one refill within six months from the first dispensing day was required, in order to exclude patients prescribed occasionally or switching to other drug classes (for example due to lack of response). 

Adherence to a single drug class was measured with the proportion of days covered (PDC) [15]. PDC was calculated as the sum of days supplied from the index date divided by the length of the observation window and then multiplied by 100. The measure was capped at 100%. 

Adherence to multiple medications was measured with the daily polypharmacy possession ratio (DPPR) [12,13]. DPPR is a relatively new measure, aimed at generalizing the calculation of the PDC over several medications. It results in the average number of medications available for each day in the observation window. As a patient may have new drugs dispensed or withdrawn at staggered time points, the calculation was performed by periods, with a period starting when a new therapeutic group began or ended. DPPR was calculated by assigning a daily score for each day of the observation window (between 0—no medication available and 1—all medications available), weighted by the number of drugs a patient should take each day. When a new drug class was added or withdrawn from the list of patients’ therapies, the daily scores of drug possession were modified accordingly, starting from the date of its addition/removal. The scores were added for each day within each period and finally divided by the number of days in the overall observation window. This value was then multiplied by 100, to obtain a percentage (%). When calculating both PDC and DPPR in the denominator, the overall observation period started at the first dispensation date and ended on 31 December 2018.

**Figure 1:**
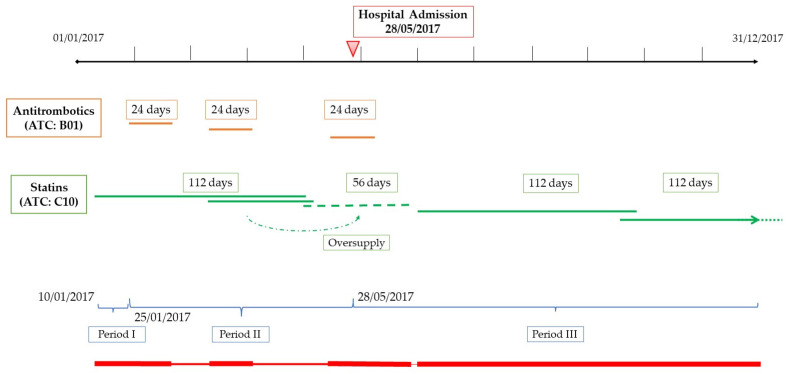
Illustrative example of the calculation of the proportion of days covered (PDC) and of the daily polypharmacy possession ratio (DPPR) for a patient prescribed with 2 medications, with 7 dispensations. The first drug was dispensed as follows: 25/01, 10/03, and 15/05 for 24 days coverage each; the second drug was dispensed as follows: 10/01 for 112 days coverage, 10/03 for 56 days coverage, 01/07, and 18/10 for 112 days coverage each. During a hospital stay on 28 May 2017, the first drug was discontinued. The dashed line indicates the carryover of excess medication from one interval to the next interval. The bottom red line indicates the DPPR daily score (the thickness is proportional to the proportion of medications available on each day: dark, 1/1 or 2/2; medium, ½; light, 0). Calculation: PDC for antithrombotics (B01): (24 + 24 + 13)/123 = 0.496 (49.6%); PDC for statins (C10): (112 + 56 + 112 + 71)/355 = 0.989 (98.9%); DPPR = [15 × 1 + (24 × 2/2 + 22 × 1/2 + 24 × 2/2 + 40 × 1/2 + 13 × 2/2) + (30 × 1 + 0 × 4 + 183 × 1)]/355 = 0.901 (90.1%).

For both PDC and DPPR, the number of days covered by each drug dispensed was calculated by dividing the total number of drugs prescribed by the defined daily dose (DDD). The main assumptions in calculating PDC and DPPR were as follows: (1) all active substances (defined by the fifth level of ATC) belonging to the same class contributed to the calculation of adherence; (2) oversupplies (excess medications) were allowed to compensate for gaps (periods in which a medication was not currently available) in the following period but not in the previous one; (3) oversupplies beyond the end of the follow-up were excluded; (4) when a patient did not receive any refill for a specific drug class after hospital discharge, the adherence for this drug class was calculated until the last hospital admission, considering this as drug discontinuation. No further adjustment was used to handle hospitalizations in the calculation of PDC and DPPR. Figure 1 shows an example of the calculation of both PDC and DPPR.

### 2.4. Statistical Analysis 

DPPR ranged from 0 to 1 (but not proportionally), and because it reached 1 (i.e., 100% adherence) in nearly 7.5% of the people, to assess factors associated with DPPR, a one-inflated beta regression model was used [16]. This is a mixture model that incorporates a beta regression model for the proportions in the interval (0, 1) and a logistic regression to model the probability mass when the proportion is 1. The results are expressed as odds ratios (ORs) with a 95% confidence interval (CI). This model was adjusted for gender, age class (<75, 75–85, >85), and the number of chronic diseases during 2016–2018 (1, 2–3, and ≥4). 

We also assessed the impact of medication adherence (DPPR) on several clinical outcomes, such as overall mortality, nursing home admissions, the number of visits to emergency departments (EDs), and the number of hospital admissions, over one year after the end of the medication adherence assessment (i.e., during 2019). The 12-month overall survival (OS) was investigated with a Cox survival regression model, and results are reported as hazard ratios (HRs) with 95%CI [16]. In addition, the crude cumulative incidence (CCI) of nursing home admission was investigated using the Fine–Grey model, to account for death as a competing risk, and the results are reported as subdistribution hazard ratios (SDHRs) with 95%CI [16]. The numbers of hospitalizations and ED visits were investigated by using a zero-inflated Poisson (ZIP) regression model [17]. In the ZIP model for count data with excess zeros, the data were assumed to be generated by a Poisson process, generating both zero and non-zero counts, and a separate process, modeled with logit regression, always generating zeros. The effect was measured as rate ratios (RRs) for the Poisson model, and as odds ratios for the logistic model, with a corresponding 95%CI. 

All models were adjusted for gender, age class, the number of chronic diseases (as above), DPPR, and participation for at least 60 days during 2018 in the healthcare program for frail patients launched by the Lombardy Region, called “Piano di Assistenza Individuale (PAI)” (individual care plan). In addition, when considering the numbers of ED visits and hospital admissions, the models were also adjusted for being hospitalized at least once in the previous period of adherence assessment. 

## 3. Results

### 3.1. Overall Patient Characteristics and Medication Adherence

**Figure 2:**
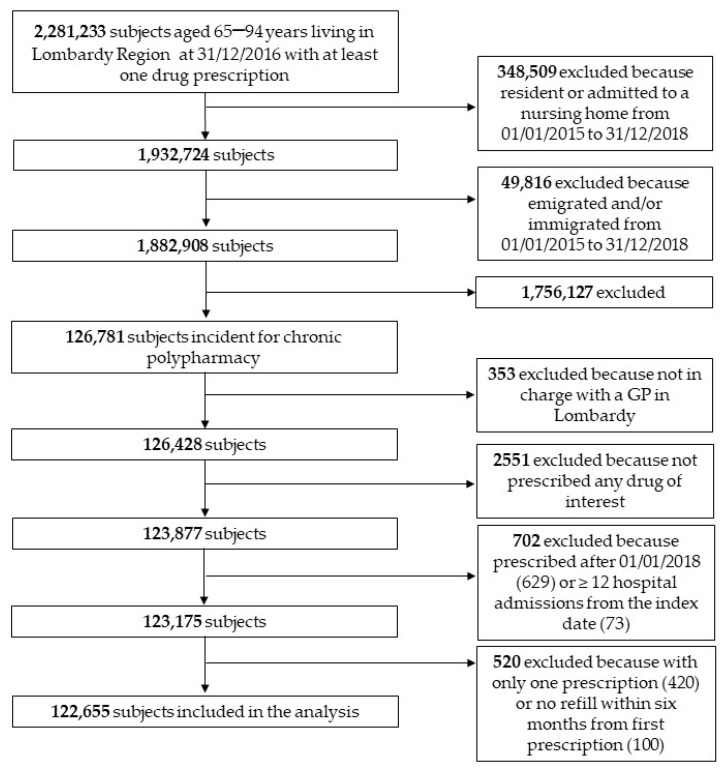
Study flow-chart.

There were 2,281,233 people aged 65–94 years living in the Lombardy Region, who received at least one medication prescription during 2016. Of these, 1,882,908 were assessable over the whole study period. In total, 126,871 were newly exposed to polypharmacy in 2016, and 123,877 took at least one of the drugs of interest. Finally, by excluding those first prescribed in 2018 and those with no refills, 122,655 people were included in the present analysis. Figure 2 shows the flowchart for the study population.

Among patients included, 64,063 (52.2%) were females, with a mean (std.dev) age of 77.3 (6.8) years (Table 1). More than half the patients were prescribed three drug classes or more (N = 71,939, 58.7%), and only 12.6% were prescribed only one.

The most prescribed drug classes were antihypertensives, followed by antithrombotics and statins (Table 1). Antihypertensives were also the class with the highest adherence level, with a mean (std.dev) PDC of 89.1% (19.4). Bisphosphonates had the lowest adherence, with a mean PDC of 76.2% (24.7). In total, 47,778 (39.0%) patients can be considered highly adherent to all their medications, because the single PDCs were ≥80%. This decreased to 30.5% for patients prescribed at least three drugs. For multiple medication adherence, the overall mean PDC was 82.4% (15.7), and the mean (std.dev) DPPR was 82.9% (15.6), with 11,898 (9.7%) patients having a DPPR of 99.5% or more. Only 3559 (29.9%) took three or more medications.

### 3.2. Factors Associated with Multiple Medication Adherence

The one-inflated beta regression (Table 2) showed that all factors were associated with multiple medication adherence. In particular, being female, aged 75 years or more, and having four or more chronic conditions were likely indicators associated with lower adherence to medications. All these factors were also associated with decreasing odds of achieving 100% adherence.

### 3.3. Clinical Outcomes

#### One-Year Mortality and Nursing Home Admission

Out of the 122,655 patients, 8760 (OS = 92.9%) died in 2019, and 2739 (CCI = 2.2%) were admitted to nursing homes the same year. The increased multiple medications adherence (i.e., DPPR) was significantly associated with better survival (HR = 0.93 for 10/100-point increase, 95%CI: 0.92–0.94) (Table 3), and with lower incidence of nursing home admissions, although with a smaller effect (Table 3). Participation in the PAI was not associated with lower mortality, but there was an association with a lower incidence of nursing home admissions, although with weak evidence (SDHR = 0.80, 95%CI: 0.65–1.01, *p* = 0.06); the wide range of 95%CI is due to the very small number of patients participating in the program.

### 3.4. Emergency Department and Hospital Admissions

In total, 65,467 (53.4%) patients visited ED at least once during 2019, accounting for 92,916 visits; of those, 50 died upon arrival, and 4506 visits were classified as “less urgent”. There were also 36,311 (29.6%) patients who were admitted at least once to the hospital.

Multiple medication adherence was associated both with a greater likelihood of no ED and hospital admission and a lower number of these (Table 4 and Table 5). Patients in the PAI were likely to have fewer hospital admissions, although this variable was not associated with the likelihood of not being admitted to the hospital (Table 5). Participation in PAI was not associated with the number of ED visits; however, these patients had a 10.3% increase in the odds of no ED visits (OR = 1.10, 95%CI: 1.01–1.21) (Table 4).

## 4. Discussion

This study investigated multiple medication adherence to the most common chronic therapies co-prescribed in a large, unselected population of community-dwelling older people exposed to chronic polypharmacy in northern Italy from 2016 to 2018, its correlations, and its effects on short-term clinical outcomes, using claims data retrieved from a healthcare administrative database. Multiple medication adherence was high, with a mean (std.dev.) DPPR of 82.9% (15.6), although with a certain degree of variability. Females, the oldest in the age group, and multimorbid patients were likely to be less adherent to multiple complex medication regimens. Multiple medication adherence influenced all clinical outcomes—mainly, overall mortality and nursing home admissions, and, although with a very small effect, welled visits and hospital admissions. The public care program PAI, aimed at improving primary care for frail patients with chronic diseases, was associated with better clinical outcomes.

Medication adherence is a crucial matter in the management of patients, especially the oldest with chronic diseases and exposed to polypharmacy, due to its impact on a patient’s health and clinical outcome. To this aim, administrative databases, often collecting data for reimbursement and not for research purposes, may represent the only economic and time-saving way to study broadly representative samples and to respond to questions regarding healthcare and services. Indeed, in Italy, administrative databases are fully representative of almost all of the population because the NHS guarantees to all citizens full or partial reimbursement for specific drug classes prescribed by GPs and bought in community pharmacies, as well as for diagnostics and laboratory testing provided by accredited providers.

Polypharmacy is a frequent condition, especially among multimorbid, older patients. Despite this fact, the literature concerning the assessment of multiple medication adherence using claims data is scarce, and only a few attempts were found [12,13,18]. Adherence to polypharmacy was generally assessed by the indices used for the single medication, with the risk of biased estimates. This research was the first to assess multiple medication adherence by using a composite measure (DPPR) in the real world, in a large cohort of elderly community-dwelling patients, through the analysis of the Lombardy Region administrative data.

We found high adherence rates, both to all the single drug classes considered (assessed by PDC) and to the same classes when co-administrated (assessed by DPPR). This is not surprising if we bear in mind that all these drug classes have been widely proved to be effective and have a strong impact on patients’ health and clinical outcomes.

Compared with our previous study [5], which assessed single medication adherence by using MPR, we found higher adherence to antidiabetics (78 vs. 70%) and antithrombotics (83 vs. 69%). The latter, in particular, could be explained by the exclusion of vitamin k antagonists, which are usually used at doses that differ on the basis of the international normalized ratio, and heparins used for the short term.

Comparing factors associated with single drug adherence, for the multiple medication regimens, we also confirmed that females, the oldest patients, and those with complex clinical pictures (≥4 chronic conditions) were likely to be less adherent to their medications [5,19]. These results can likely be explained by the fact that women spend less time taking care of themselves, in favor of family members. Among the elderly with multimorbidity and exposed to polypharmacy, difficulties in managing a complex medication regimen, concerns about side effects, and forgetfulness may well explain the poor medication adherence [20].

Assessing adherence to multiple medication regimens with composite measures allowed us to investigate their impact on several clinical outcomes of interest. In this study, multiple medication adherence was associated with a lower mortality rate and a lower incidence of nursing home admissions and with small although significant effects for ED and hospital admissions. As concerns mortality, the rates are in line with previous research assessing adherence to single-class medication [21,22,23,24]. However, no studies have reported the association between medication adherence and the other outcomes considered in older populations in primary care.

We previously demonstrated that participation in the regional program for older patients with multiple chronic diseases and polypharmacy was associated with better adherence to all the chronic therapies [5].

Herein, we showed that this participation results in a greater likelihood of not being admitted to a nursing home, although with borderline statistical significance, no ED visit, and a lower number of hospital admissions. This confirms that better and deeper clinical management by GPs, who are the most representative stakeholders involved in this program, improves patients’ clinical outcomes.

### Strengths and Limitations

Several strengths need to be mentioned. The drug prescription database provides accurate data because pharmacists are required to report prescriptions in detail to obtain reimbursement, and incorrect reports have legal consequences and lead to the lack of reimbursement. Second, since the drugs analyzed are free or almost free of charge but only if dispensed against prescriptions, the amounts prescribed outside the NHS database are very small, except for aspirin, due to its very low cost. The main limitations concern the lack of indication for drug use and dosages. We, therefore, had to resort to the use of DDD, which may have strongly influenced the calculation of PDC and DPPR. However, the therapeutic dose prescribed was often not specified in many administrative databases, so the use of DDD may facilitate national and international comparisons of drug consumption. We may also have overestimated both single PDCs and the DPPR because, in calculations, we included all active substances at the fifth ATC level, thus overlooking possible overlaps of drugs belonging to the same class. 

Finally, data for this study refer to the last years of the second decade of 2000. More recent analysis may provide biased results. Indeed, the COVID-19 pandemic occurred in 2020, and its continuation until now has likely affected the relationship among GPs and their patients, especially the oldest ones. GPs had to manage patient requests remotely, using information technology systems available and, consequently, may have changed their prescription patterns, prescribing much more medications altogether and less frequently over time, thus making results of such analysis based on claims data unreliable.

## 5. Conclusions

In the period of 2016–2018, there was high adherence to multiple drug regimens of the five classes most prescribed for chronic diseases in community-dwelling older people living in the Italian Lombardy Region, and this was associated with better patient clinical outcomes.

The daily polypharmacy possession ratio (DPPR) allowed us to calculate adherence to the combination of all the chronic drugs used, available to hand each day, for this large population of multimorbid community-dwelling older people exposed to polypharmacy, on the basis of data from an administrative database. This composite measure was useful to assess the overall effect of multiple medication adherence on patients’ clinical outcomes. This method could, therefore, support policymakers and stakeholders in checking the important issue of medication adherence in the older population, where polypharmacy is the main pharmacological pattern.

## Figures and Tables

**Table 1:** Patients’ characteristics.

VARIABLE			N (%)
**SEX**			
	Male		58,592 (47.8)
	Female		64,063 (52.2)
**AGE**			
	Median (IQR)	77 (72–82)	
	65–69		19,495 (15.9)
	70–74		24,588 (20.0)
	75–79		31,999 (26.1)
	80–84		26,695 (21.8)
	85–89		15,390 (12.5)
	90–94		4488 (3.7)
**PAI**			5036 (4.1)
**HOSPITAL ADMISSIONS**			
	0		66,574 (54.3)
	1		26,127 (21.3)
	2		14,350 (11.7)
	3		7287 (5.9)
	≥4		8317 (6.8)
**NUMBER OF CHRONIC DISEASES**	Median (IQR)	2 (2–3)	
**NUMBER OF DRUGS**			
	1		15,466 (12.6)
	2		35,250 (28.7)
	3		49,644 (40.5)
	4		21,579 (17.6)
	5		716 (0.6)
**DRUG CATEGORY**			
	Drugs used for diabetes		39,730 (32.1)
	Antithrombotic agents		85,404 (69.6)
	Antihypertensives		101,804 (83.0)
	Statins		84,915 (69.2)
	Bisphosphonates		12,941 (10.6)

Legend: PAI: “Piano di Assistenza Individuale” (individual care plan), IQR: interquartile range.

**Table 2:** One-inflated beta regression model for assessing factors associated with multiple medication adherence (DPPR) (odds ratios and corresponding 95% confidence intervals).

	BETA REGRESSION	ONE INFLATION
	OR (95%CI)	OR (95%CI)
**SEX**		
**MALE**	1	1
**FEMALE**	0.85 (0.84–0.86)	0.82 (0.78–0.85)
**AGE GROUPS**		
**>65**	1	1
**>75**	0.87 (0.86–0.88)	0.86 (0.82–0.90)
**>85**	0.77 (0.76–0.79)	0.90 (0.84–0.96)
**NUMBER OF DISEASES**	1	1
**1**		
**2/3**	0.96 (0.94–0.97)	0.68 (0.65–0.72)
**4+**	0.88 (0.86–0.90)	0.57 (0.53–0.61)

Legend: OR: odds ratio, CI: confidence interval.

**Table 3:** Cox proportional hazard model on 12-month mortality (hazard ratios with 95% confidence intervals), and Fine–Grey model for the incidence of nursing home admission (subdistribution HRs with 95%CI).

	SURVIVAL	NURSING HOME ADMISSION
	HR (95%CI)	SDHR (95%CI)
**SEX**		
**MALE**	1	1
**FEMALE**	0.74 (0.70–0.77)	1.38 (1.27–1.50)
**AGE GROUPS**		
**>65**	1	1
**>75**	2.20 (2.08–2.34)	3.64 (3.18–4.16)
**>85**	5.85 (5.49–6.23)	9.51 (8.30–10.91)
**NUMBER OF DISEASES**		
**1**	1	1
**2/3**	1.39 (1.29–1.49)	0.98 (0.88–1.08)
**4+**	2.51 (2.33–2.70)	1.30 (1.16–1.47)
**PAI**		
**NO**	1	1
**YES**	0.94 (0.83–1.05)	0.80 (0.65–1.01)
**DPPR**	0.93 (0.92–0.94)	0.95 (0.93–0.97)

Legend: HR: hazard ratio; SDHR: subdistribution hazard ratio; CI: confidence interval; PAI: “Piano di Assistenza Individuale” (individual care plan), DPPR: daily polypharmacy possession ratio.

**Table 4:** Zero-inflated Poisson regression model for factors associated with the number of emergency department visits (rate ratios and corresponding 95%CI (Poisson regression); odds ratios and corresponding 95%CI (zero inflation)).

	POISSON	ZERO INFLATION
	RR (95%CI)	OR (95%CI)
**SEX**		
**MALE**	1	1
**FEMALE**	0.94 (0.92–0.96)	0.88 (0.84–0.91)
**AGE GROUPS**		
**>65**	1	1
**>75**	1.04 (1.02–1.06)	0.71 (0.68–0.73)
**>85**	1.04 (1.02–1.07)	0.51 (0.45–0.57)
**NUMBER OF DISEASES**	1	1
**1**		
**2/3**	1.09 (1.06–1.12)	0.95 (0.90–1.01)
**4+**	1.24 (1.21–1.29)	0.74 (0.69–0.79)
**DPPR (10 POINT)**	0.98 (0.97–0.99)	1.02 (1.01–1.03)
**PAI**		
**NO**	1	1
**YES**	0.97 (0.92–1.01)	1.10 (1.01–1.21)
**PREVIOUS ED VISITS**		
**NO**	1	1
**YES**	1.29 (1.26–1.31)	0.69 (0.66–0.71)

Legend: OR: odds ratio, RR: rate ratio; CI: confidence interval, DPPR: daily polypharmacy possession ratio, ED: emergency department; PAI: “Piano di Assistenza Individuale” (individual care plan).

**Table 5:** Zero-inflated Poisson regression model for assessing factors associated with the number of hospital admissions (rate ratio and corresponding 95%CI (Poisson regression); odds ratios and corresponding 95%CI (zero inflation)).

	POISSON	ZERO INFLATION
	RR (95%CI)	OR (95%CI)
**SEX**	1	1
**MALE**		
**FEMALE**	0.94 (0.91–0.96)	1.16 (1.11–1.22)
**AGE GROUPS**		
**>65**	1	1
**>75**	0.97 (0.95–1.00)	0.79 (0.76–0.83)
**>85**	0.84 (0.81–0.88)	0.51 (0.47–0.55)
**NUMBER OF DISEASES**		
**1**	1	
**2/3**	1.11 (1.06–1.17)	0.85 (0.79–0.91)
**4+**	1.28 (1.21–1.33)	0.62 (0.57–0.67)
**DPPR (10 POINT)**	0.99 (0.98–1.00)	1.03 (1.01–1.04)
**PAI**		
**NO**	1	1
**YES**	0.89 (0.83–0.96)	0.98 (0.87–1.11)
**PREVIOUS HOSPITAL ADMISSIONS**		
**NO**	1	1
**YES**	1.32 (1.28–1.36)	0.49 (0.46–0.51)

Legend: OR: odds ratio, RR: rate ratio; CI: confidence interval; DPPR: daily polypharmacy possession ratio, PAI: “Piano di Assistenza Individuale” (individual care plan).

## Data Availability

Research data are not shared.
